# Dynamic alterations in the amplitude of low-frequency fluctuation in patients with cerebral small vessel disease

**DOI:** 10.3389/fnmol.2023.1200756

**Published:** 2023-09-22

**Authors:** Jiarui Song, Ting Lei, Yajun Li, Lijing Zhou, Wei Yan, Haiqing Li, Li Chen

**Affiliations:** ^1^Department of Radiology, Affiliated Hospital of North Sichuan Medical College, Nanchong, China; ^2^Department of Nuclear Medicine, Chongqing Liangjiang New District people’s Hospital, Chongqing, China

**Keywords:** cognition, cerebral small vessel disease, magnetic resonance imaging, dynamic amplitude of low-frequency fluctuation, memory

## Abstract

**Background and purpose:**

Previous studies have focused on the changes of dynamic and static functional connections in cerebral small vessel disease (CSVD). However, the dynamic characteristics of local brain activity are poorly understood. The purpose of this study was to investigate the dynamic cerebral activity changes in patients with CSVD using the dynamic amplitude of low-frequency fluctuation (d-ALFF).

**Methods:**

A total of 104 CSVD patients with cognitive impairment (CSVD-CI, *n* = 52) or normal cognition (CSVD-NC, *n* = 52) and 63 matched healthy controls (HCs) were included in this study. Every participant underwent magnetic resonance imaging scans and a battery of neuropsychological examinations. The dynamics of spontaneous brain activity were assessed using dynamic changes in the amplitude of low-frequency fluctuation (ALFF) with the sliding-window method. We used voxel-wise one-way analysis of variance (ANOVA) to compare dynamic ALFF variability among the three groups. Post-hoc *t*-tests were used to evaluate differences between each group pair. Finally, the brain regions with d-ALF*F* values with differences between CSVD subgroups were taken as regions of interest (ROI), and the d-ALFF values corresponding to the ROI were extracted for partial correlation analysis with memory.

**Results:**

(1) There was no significant difference in age (*p* = 0.120), sex (*p* = 0.673) and education (*p* = 0.067) among CSVD-CI, CSVD-NC and HC groups, but there were significant differences Prevalence of hypertension and diabetes mellitus among the three groups (*p* < 10^−3^). There were significant differences in scores of several neuropsychological scales among the three groups (*p* < 10^−3^). (2) ANOVA and post-hoc *t-*test showed that there were dynamic abnormalities of spontaneous activity in several brain regions in three groups, mainly located in bilateral parahippocampal gyrus and bilateral hippocampus, bilateral insular and frontal lobes, and the static activity abnormalities in bilateral parahippocampal gyrus and bilateral hippocampal regions were observed at the same time, suggesting that bilateral parahippocampal gyrus and bilateral hippocampus may be the key brain regions for cognitive impairment caused by CSVD. (3) The correlation showed that d-ALFF in the bilateral insular was slightly correlated with the Mini-Mental State Examination (MMSE) score and disease progression rate. The d-ALFF value of the left postcentral gyrus was negatively correlated with the Clock Drawing Test (CDT) score (*r* = −0.416, *p* = 0.004), and the d-ALFF value of the right postcentral gyrus was negatively correlated with the Rey’s Auditory Verbal Learning Test (RAVLT) word recognition (*r* = −0.320, *p* = 0.028).

**Conclusion:**

There is a wide range of dynamic abnormalities of spontaneous brain activity in patients with CSVD, in which the abnormalities of this activity in specific brain regions are related to memory and execution or emotion.

## 1. Introduction

Cerebral small vessel disease (CSVD) is a group of cerebrovascular diseases with similar clinical symptoms and imaging manifestations caused by the injury of small blood vessels, Du et al. (2007), Kim et al. (2014), Mollink et al. (2019) such as the short cortical artery, long medulla artery, lenticular artery, Heubner recurrent artery, perforating colliculus artery, and perforating branch artery of the brainstem (Werring et al., 2004; Tao et al., 2019). CSVD is an important vascular factor causing dementia. Previous research has shown that vascular cognitive impairment caused by CSVD varies from mild cognitive impairment (MCI) to vascular dementia (VaD) (Liu et al., 2018; Ter Telgte et al., 2018). MCI is also considered to be the first cognitive manifestation of Alzheimer’s disease (AD) (Liu et al., 2018; Ter Telgte et al., 2018; Tao et al., 2019; Wang J. et al., 2019). The imaging findings of CSVD include recent small subcortical infarcts (RSSI), lacune of presumed vascular origin (LA), white matter hyperintensity of presumed vascular origin (WMH), perivascular space (PVS), cerebral microbleeds (CMB), and brain atrophy (Ter Telgte et al., 2018). CSVD may cause cognitive disorders, execution disorders, gait instability, emotional disorders, sleep disorders, urinary incontinence, and other clinical symptoms, and affect patients’ memory, language, attention, visual space function, and more (Ter Telgte et al., 2018; Frey et al., 2019; Kim et al., 2020; Chojdak-Łukasiewicz et al., 2021). At present, the pathophysiological mechanisms of CSVD are not completely clear, and there is no characteristic CSVD diagnostic tool, so it is necessary to look for objective indicators to help diagnose CSVD.

Resting-state functional magnetic resonance imaging (rs-fMRI) is a non-invasive neuroimaging technique that has been widely used to study the spontaneous brain *in vivo* (Liu et al., 2019; Zhou et al., 2020). The amplitude of low-frequency fluctuation (ALFF) is a common method to describe the regional characteristics of rs-fMRI data, which can infer the level of neural tissue activity in a resting state from the point of view of energy (Zang et al., 2007; Li et al., 2014). In CSVD patients with CMB, the ALFF values in the right insular, putamen, and left pre-cuneate gyrus were significantly increased, while the ALFF values in the right precentral and postcentral gyri were significantly decreased (Feng et al., 2021). In patients with leukoaraiosis, the ALFF in the cingulate gyrus/cuneus was significantly decreased, while the ALFF in the temporal region was significantly increased (Wang P. et al., 2019). There were significant differences in ALFF values of the bilateral parahippocampal gyrus, right putamen, right middle frontal gyrus, left cuneate lobe, and bilateral prefrontal lobe in patients with depression and MCI (Li et al., 2017). Another study showed that the ALFF decreased in the bilateral anterior cingulate lobe, while the ALFF increased in the bilateral anterior cingulate cortex, left insular, and hippocampus, which was significantly correlated with cognitive ability (Li et al., 2014). In patient with WMH and cognitive impairment (CI),we found there is a mismatch between the WMH burden and cognitive status (Prins and Scheltens, 2015), WMH with CT showed spontaneous brain activity damage. The abnormality of ALFF value was related to WMH related CI, and the abnormal ALFF value was an effective index to identify WMHs related CI (Ni et al., 2022). However, these studies have relied on an implicit assumption that brain activity remains constant during rs-fMRI scans and cannot reflect the temporal dynamics of the internal brain activity of patients (Tagliazucchi et al., 2014; Tomasi et al., 2016; Cui et al., 2020). With the progress of technology, more and more studies have shown that in a resting state, the inherent activity of the brain still changes with time. The dynamic ALFF (d-ALFF), an indicator of the variance of ALFF, is an effective tool to explore brain dynamics in generalized anxiety disorder (Cui et al., 2020), depression (Golestani et al., 2017), Alzheimer’s disease (Lin et al., 2021), Parkinson’s (Lin et al., 2015), amyotrophic lateral sclerosis (Ma X. et al., 2020), diabetes (Huang et al., 2021), and cervical spondylosis (Ma M. et al., 2020).

To date, there are few studies that have evaluated the combined effects of d-ALFF in patients with CSVD. Hence, in the present rs-fMRI study, we applied a combination of ALFF with the sliding window technique to compare d-ALFF alterations between CSVD patients with and without cognitive impairment (CI). This study aimed to determine the functional brain abnormalities caused by CSVD using the static and d-ALFF methods to eventually identify potential targets to improve the present understanding and treatment strategies for CSVD. We hypothesized that (1) CSVD patients would show significant functional brain changes in several brain regions compared with controls and (2) alterations in spontaneous brain activity would be related to clinical parameters in CSVD patients.

## 2. Materials and methods

### 2.1. Participants

The present study was approved by the ethics committee of our hospital. In total, 167 right-handed participants, including 104 patients meeting the criteria of CSVD and 63 healthy controls (HCs), were recruited in the study.

The patients with CSVD met the following criteria (Román et al., 2002): (1) white matter lesions with hyperintensities extending periventricularly and deep white matter; extending caps (>10 mm as measured parallel to ventricle) or irregular halo (>10 mm with broad, irregular margins and extending into deep white matter); and diffusely confluent hyperintensities (>25 mm, irregular shape) or extensive white-matter change (diffuse hyperintensity without focal lesions); (2) lacunar cases with multiple lacunas (>5) in the deep gray matter and at least moderate white-matter lesions; (3) absence of hemorrhages, cortical and territorial infarcts and watershed infarcts; signs of normal pressure hydrocephalus; and specific causes of white-matter lesions.

CSVD-CI was diagnosed according to the following criteria (Galluzzi et al., 2005): (1) in accordance with the criteria of CSVD; (2) subjective cognitive complaints reported by the participant or his/her caregiver; (3) objective cognitive impairments, although not meeting the Diagnostic and Statistical Manual of Mental Disorders, fourth edition (DSM-5) criteria for dementia; (4) a Clinical Dementia Rating Scale (CDR) score = 0.5; (5) a Mini-Mental State Examination (MMSE) score of 24–26.

The CSVD-NC group met the following criteria: (1) in accordance with the criteria of CSVD; (2) normal daily life activities and cognitive assessments; (3) a CDR score = 0; (4) a MMSE score ≥ 27.

The criteria for the normal controls (HCs) were: (1) no neurological and psychiatric disorders; (2) no abnormal findings on conventional brain MRI; (3) no cognitive complaints.

The exclusion criteria for all of the subjects included the following: (1) metabolic conditions, such as hypothyroidism or folic acid deficiencies; (2) psychiatric disorders, such as schizophrenia or depression; (3) Parkinsonian syndrome, epilepsy, and other nervous system diseases that can influence cognitive function.

### 2.2. Neuropsychological assessment

All participants underwent a comprehensive neuropsychological battery. The Activities of Daily Living scale (ADL) and CDR were used to evaluate daily life capacities. The Hamilton anxiety scale (HAM-A) and Hamilton depression scale (HAMD) was used to exclude those with potential depression. The five cognitive variables included in the present analysis are: (1) Global cognition, which was measured by the MMSE; (2) Episodic memory, which was evaluated by the RAVLT; (3) Visuospatial perception, which was assessed by the CDT; (4) Language function, which was examined by the Boston Naming Test (BNT); (5) Executive function and working memory, which were assessed by Trail Making Tests (TMT-A and TMT-B) and the Stroop Test (color-word).

### 2.3. Data acquisition

ALL MR images were acquired on a 3.0 T GE Medical Systems MR medicine (Discovery MR750, United States) with a standard 32-channel head coil. Foam padding was used to restrict head movement, and ear plugs were used to minimize the scanner noise. The subjects were told to relax, keep their eyes closed, and remain awake. Rs-fMRI data were acquired using a Gradient recalled echo–echo planar imaging (GRE-EPI) sequences with the following parameters:: repetition time (TR) = 2,000 ms, echo time (TE) = 40 ms, flip angle = 90°, thickness = 4.0, and no gap, field of view (FOV) = 240 mm × 240 mm, and matrix = 64 × 64. A total of 240 volumes were obtained in 8 min. Evaluation of CSVD-related pathology was observed on T2 fluid-attenuated inversion recovery (FLAIR)–weighted images: TR = 8,000 ms, TE = 126 ms, inversion time (TI) = 2,100 ms, thickness = 3 mm, FOV = 240 mm × 240 mm, and matrix = 256 × 192.

### 2.4. Data preprocessing

Rs-fMRI images were preprocessed using the Data Processing and Analysis of Brain Imaging (DPABI) toolbox.^1^ The preprocessing steps included: (a) Convert image to NIFITdata format; (b) Delete the first 10 time points in the subject’s scanning time series to ensure that the scan data are obtained when the subject is in a steady state; (c) slice timing correction for the remaining 230 fMRI images; (d) head motion correction: Some slight head movements caused by physiological activities such as breathing and heartbeat were corrected, and those with head translation >3 mm and/or rotation >3 °were excluded; (e) Spatial standardization and smoothing: the functional images of the subjects were registered to the standard space (MNI space) made by the Montreal Institute of Neuroscience, and resampled to 3 mm × 3 mm × 3 mm voxel size. Then, 6 mm full width and half height (full width at half maxima, FWHM) Gaussian smoothing is used to check the functional image after spatial standardization for spatial smoothing; (f) detrending. (g) Removal of covariates and linear drift: multiple regression method was used to remove 24 covariates such as cephalo metric parameters, white matter and cerebrospinal fluid to reduce the effects of respiration and heartbeat, and global signal regression (Global signal regression, GSR) processing was performed. (h) Fliter: Using bandwidth 0.01–0.08 Hz to filter data; (i) motion scrubbing to remove the “bad” time points and their 1-back and 2-forward time points on the basis of a FD threshold of 0.5 mm.

### 2.5. Dynamic ALFF and static ALFF computation

The d-ALFF of patients were evaluated using Uses a MATLAB-based software called Dynamic BC toolbox, and dynamic analysis was carried out using the sliding window method (Liao et al., 2014). According to previous research, the time window length was set to 50TR (100 s) and the step size was set to 5TR (10 s) (Cui et al., 2020). Window length is an important parameter in the calculation of resting state dynamics. According to previous studies, the minimum window length should not be less than 1/*f*_min_ (minimum frequency of time series), because a shorter window length may increase the risk of false fluctuations, and a longer window will lose its dynamics to a certain extent. The window width was set to 50 TR and the step size was set to 5TR so that each time window contained 50 time points out of all time points in the time series, and the start point of each time window was 5TR later than the start point of the previous time window. The time series of each participant was divided into 36 windows, and the ALFF map was computed within each window, generating a set of ALFF maps for each participant. Subsequently, we measured the variance of these maps using SD to evaluate the temporal variability of d-ALFF. Finally, for each participant, the d-ALFF variability of each voxel was further transformed into z-scores by subtracting the mean and dividing by the SD of global values. Finally, for each participant, the d-ALFF variability of each voxel was further transformed into a *z*-score by subtracting the mean and dividing by the SD of global values.

DPABI software and SPM12 are used to calculate the ALFF value. The specific steps for calculating the ALFF value were as follows: (1) fast Fourier transform (FFT) was performed on the preprocessed data, and the time series of each voxel was transformed into the frequency domain and then filtered (the power spectrum of each voxel in the range of 0.01–0.08 Hz is obtained by eliminating high-frequency respiratory, heartbeat, and low-frequency noise by bandpass filtering in the 0.01–0.08 Hz band); (2) the square root of each frequency power spectrum was calculated; (3) the average square root of the power spectrum (ALFF value) was calculated, and the Fisher transform was used to convert all subjects’ ALFF values into *z*-scores; (4) the resulting values (FWHM = 6 mm) were smoothed for statistical analyses.

### 2.6. Statistics

#### 2.6.1. Statistical analysis of demographic data and clinical scale

IBM SPSS25.00 (IBM SPSS Inc., Chicago, IL, United States) software was used for statistical analysis. The demographics, clinical data, and neuropsychological scale of the three groups were compared by analysis of variance (ANOVA), and the gender was compared using a chi-square test. Furthermore, a *post hoc* t test was used to detect the differences among groups. All data were analyzed using a Kolmogorov–Smirnov test for normality. All statistical tests were double-tailed, and a *p* < 0.05 was statistically significant.

#### 2.6.2. Statistical analysis of imaging data

Using age, sex, and years of education as interference variables, an ANOVA was used to compare the differences of d-ALFF among the three groups (CSVD-NC, CSVD-CI, and HC groups). Multiple comparisons were corrected using a Family Wise Error correction (FWE) based on cluster level with an initial vertex-wise threshold of *p* < 0.001. The Extract regions of interest (ROI) signals of REST software was used to extract the dynamic/static low-frequency amplitudes of different brain regions among the CSVD-CI, CSVD-NC, and HC groups.

#### 2.6.3. Correlation analysis

Finally, partial correlation analyses was used to examine the relationship between the brain regions with inter-group and intra-group differences in d-ALFF and the neuropsychological scale, and age, sex, and years of education were taken as regression variables. Calculate *p*-value and partial correlation coefficient (*r*-value). A *p* < 0.05 was considered statistically significant.

## 3. Results

### 3.1. Demographic and clinical characteristics

The subjects’ demographics, clinical data, and some neuropsychological data are shown in Table 1. There was no significant difference in sex (*p* = 0.758), age (*p* = 0.120), years of education (*p* = 0.063), smoking history (*p* = 0.578), and drinking history (*p* = 0.104). The prevalence rates of systolic blood pressure (*p* < 10^−3^) and hypertension (*p* < 10^−3^) in CSVD patients were higher than those in the HC group. There was a significant difference in the prevalence of diabetes among the three groups (*p* < 10^−3^). The prevalence rate of CDVD-CI was higher than that of the HC group, and the prevalence rate of diabetes in the CSVD-CI group was higher than that in the CSVD-NC group (*p* < 10^−3^). Regarding mood, the patients in the CSVD-CI displayed significantly higher HAM-A scores (indicating higher levels of anxiety) than those in the CSVD-NC and HC group (*p* < 10^−3^).However, the score of the HAM-D show no significantly difference between three group.

**Table 1:** Comparisons of demographic and clinical data of the CSVD-CI, CSVD-NC and HC groups.

	CSVD-CI (*n* = 52)	CSVD-NC (*n* = 52)	HC (*n* = 63)	*p*-value
Gender (M/F)	31/21	19/33	30/32	0.062
Age (year)	69.63 ± 5.75	69.12 ± 5.01	67.62 ± 5.56	0.120
Education (year)	9.21 ± 2.00^a^	10.37 ± 2.70^a^	9.79 ± 2.71	0.067
Systolic (mmhg)	147.6 ± 19.73^a^	146.27 ± 20.29^a^	128.67 ± 14.13	< 10^−3^
Diastolic (mmHg)	80.42 ± 9.82	81.76 ± 10.62	78.33 ± 8.65	0.162
*History*				
hypertension	73.1%^a^	65.3%^a^	25.3%	< 10^−3^
Diabetes Mellitus	23.0%^b^	21.1%	19.0%	< 10^−3^
Smoking	25.0%	21.1%	19.0%	0.743
Drinking	17.3%	25.0%	12.7%	0.232
HAM-A	3.88 ± 1.90^a^^,^^b^	2.88 ± 2.38	2.27 ± 1.95	< 10^−3^
HAM-D	2.87 ± 2.15	2.76 ± 1.73	2.14 ± 2.12	0.150

One-way ANOVA and post-hoc analyses were used to assess differences in the continuous variables across the groups. The Kruskal–Wallis *H* was used for the nonparametric data, and the *χ*^2^ test was used for the sex proportions. The data are presented as the mean ± SD for normally distributed variables.

a Significant compared to the HC group (*p* < 0.05).

b Significant compared to the CSVD-NC (*p* < 0.005); HC, health controls; CSVD-NC, cerebral small-vessel Disease without cognitive impairments, CSVD-CI cerebral small-vessel Disease with cognitive impairments.

As shown in Table 2, there were significant differences in the RAVLT scores, including immediate recall, delayed recall, and word recognition, among the three groups (*p* < 10^−3^). The immediate recall and delayed recall in the CSVD-CI group were significantly lower than in the CSVD-NC and HC groups (*p* < 10^−3^), and the word recognition test in the CSVD-CI group was lower than in the CSVD-NC group (*p* < 10^−3^). The BNT and CDT scores in the CSVD group were significantly lower than in the HC group (*p* < 10^–3^). The TMT test scores in patients with CSVD were significantly lower than in HCs (*p* < 10^−3^), and the duration of the TMT test in patients with CSVD-CI was significantly longer than that in CSVD-NC.

**Table 2:** Results of Neuropsychological Tests in the CSVD-CI, csvd-NC and HC groups.

	CSVD-CI (*n* = 52)	CSVD-NC (*n* = 52)	HC (*n* = 63)	*F*/*p*-value
MMSE	22.58 ± 4.19^a^^,^^b^	28.02 ± 1.20	27.92 ± 1.56	76.26/<10^−3^
RAVLT				
Immediate recall	4.19 ± 1.95^a^^,^^b^	7.03 ± 2.00	7.69 ± 1.44	59.33/<10^−3^
Delay recall (5 min)	3.92 ± 2.63^a^^,^^b^	7.33 ± 2.81^a^	9.03 ± 2.19	58.80/<10^−3^
Delay recall (20 min)	3.42 ± 2.54^a^^,^^b^	7.23 ± 2.70^a^	8.62 ± 2.37	63.20/<10^−3^
Recognition	13.49 ± 7.10^b^	18.85 ± 6.72^a^	15.83 ± 5.17	9.34/<10^−3^
BNT	18.94 ± 4.91^a^^,^^b^	23.38 ± 4.14	23.02 ± 3.89	17.47/<10^−3^
CDT	2.25 ± 1.31^a^^,^^b^	3.20 ± 1.15	3.21 ± 0.77	13.87/<10^−3^
TMT-A(s)	169.88 ± 75.19^a^^,^^b^	107.95 ± 46.20^a^	80.88 ± 24.38	43.73/<10^−3^
TMT-B(s)	373.38 ± 132.66^a^^,^^b^	254.26 ± 93.29^a^	179.16 ± 76.10	51.98/<10^−3^

One-way ANOVA and *post-hoc* analyses were used to assess differences in the continuous variables across the groups. The data are presented as the mean value ± SD.

a Significant compared to the HC group (*p* < 0.05).

b Significant compared to the CSVD-NC group (*p* < 0.05); MMSE, Mini-Mental State Examination; RAVLT, Rey Auditory Verbal Learning Test; BNT, Boston Naming Test; CDT, Clock Drawing Test; TMT, Trail Making Test.

### 3.2. Differences in dynamic ALFF

**Figure 1:**
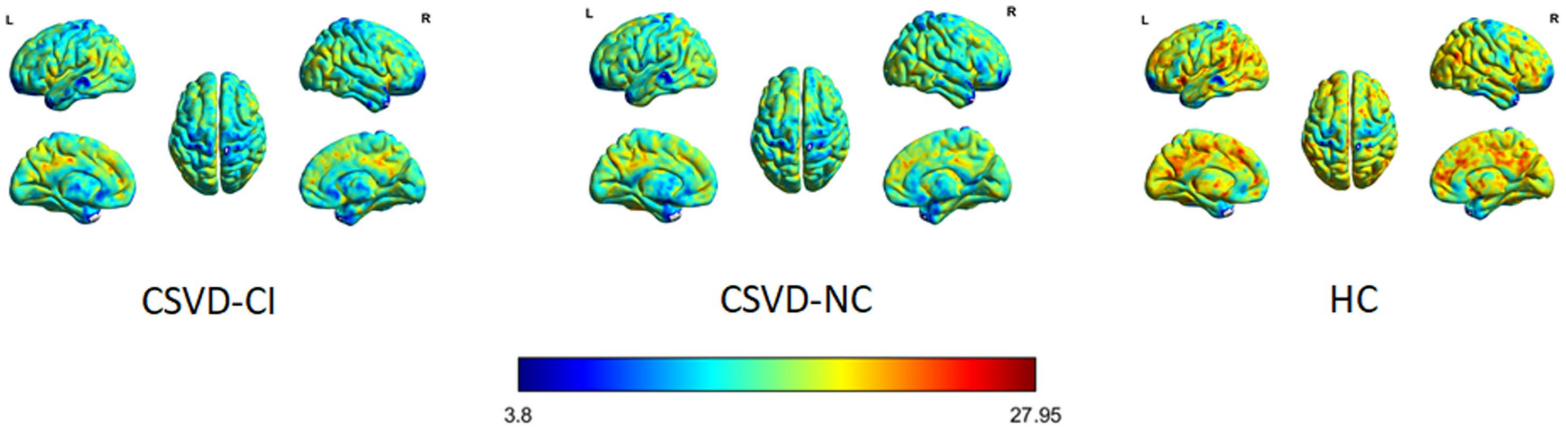
HC, healthy control; CSVD-NC, cerebral small-vessel Disease without cognitive impairment, CSVD-CI, cerebral small-vessel Disease with cognitive impairment.

**Figure 2:**
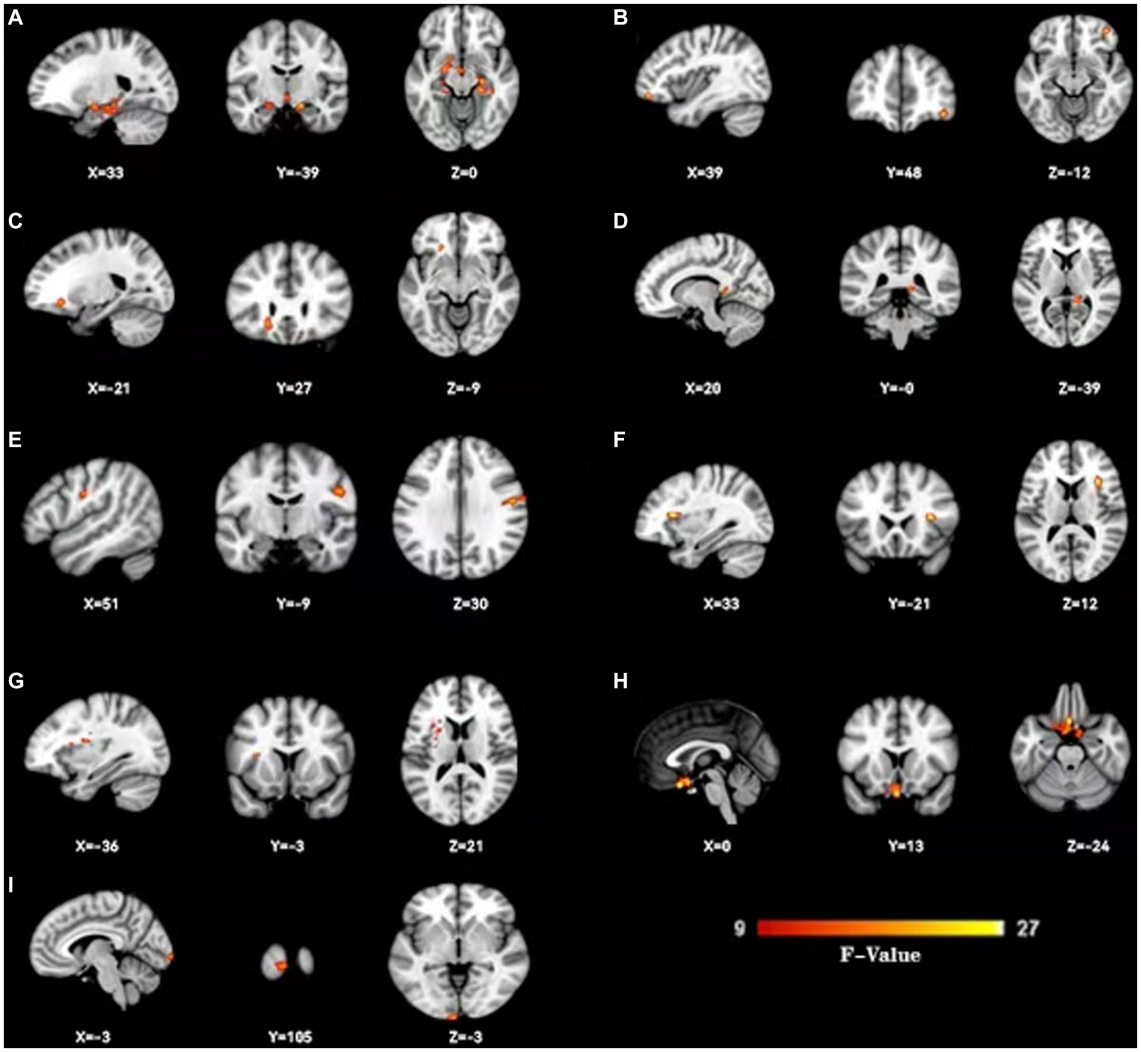
Change dynamic ALFF among three groups. Three groups show dynamic ALFF (d-ALFF) widespread differences were present predominantly in the bilateral hippocampus and parahippocampal gyrus **(A)**,right orbital middle frontal gyrus **(B)**, left orbital inferior frontal gyrus **(C)**, right wedge prefrontal gyrus **(D)**, right postcentral gyrus **(E)**, right insular **(F)**, left insular **(G)**,left straight gyrus **(H)** and left taloid cortex **(I)**.

**Figure 3:**
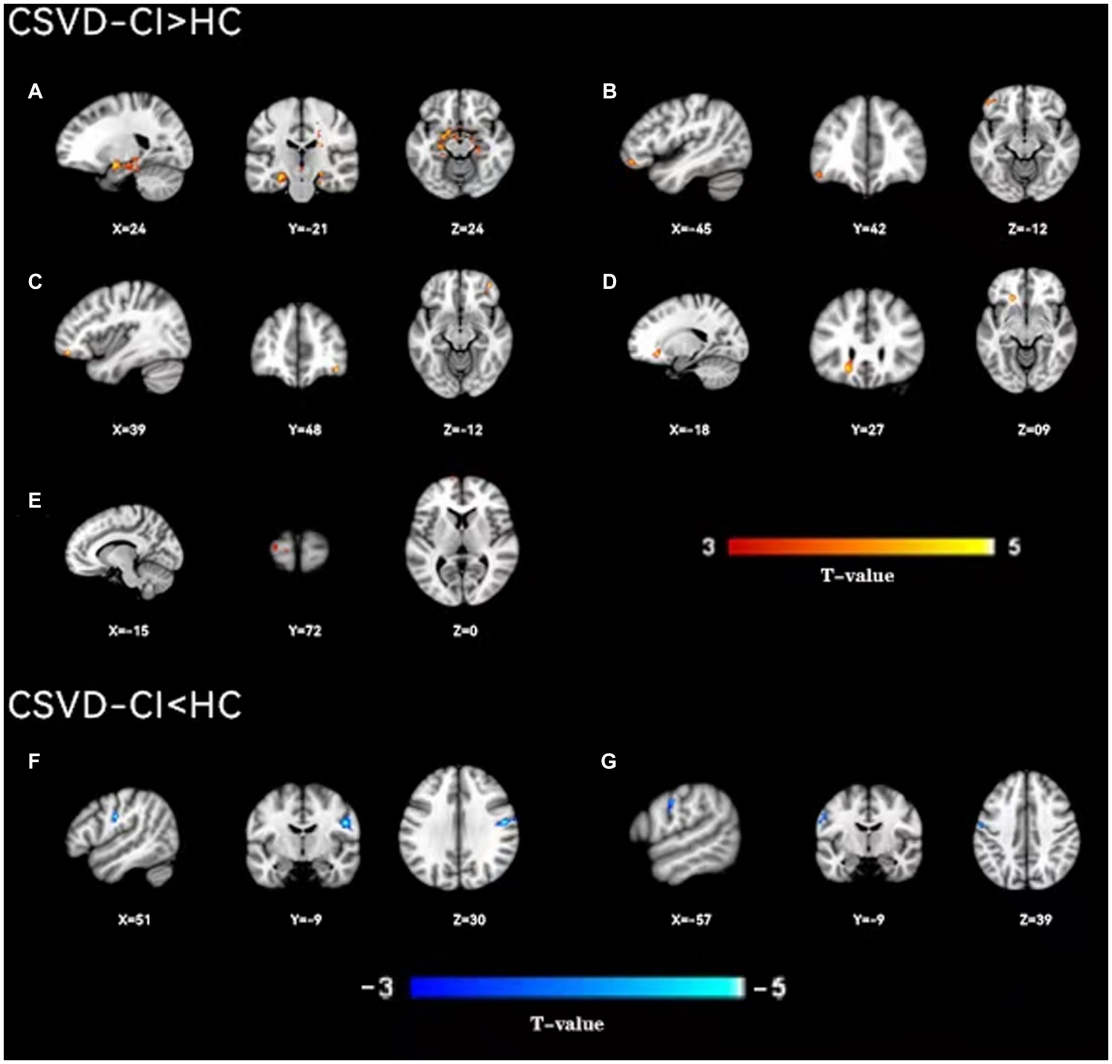
A two-sample *t*-test was used to compare the dynamic ALFF maps between CSVD-CI patients and healthy controls. Significantly increased dynamic ALFF in Bilateral hippocampus and parahippocampal gyrus **(A)**, left orbital inferior frontal gyrus **(B)**, right orbital middle frontal gyrus **(C)**, left orbital middle frontal gyrus **(D)**, left dorsolateral supraorbital gyrus **(E)**, significantly decreased dynamic ALFF in Bilateral postcentral gyrus **(F)**, right postcentral gyrus **(G)**, left postcentral gyrus.

**Figure 4:**
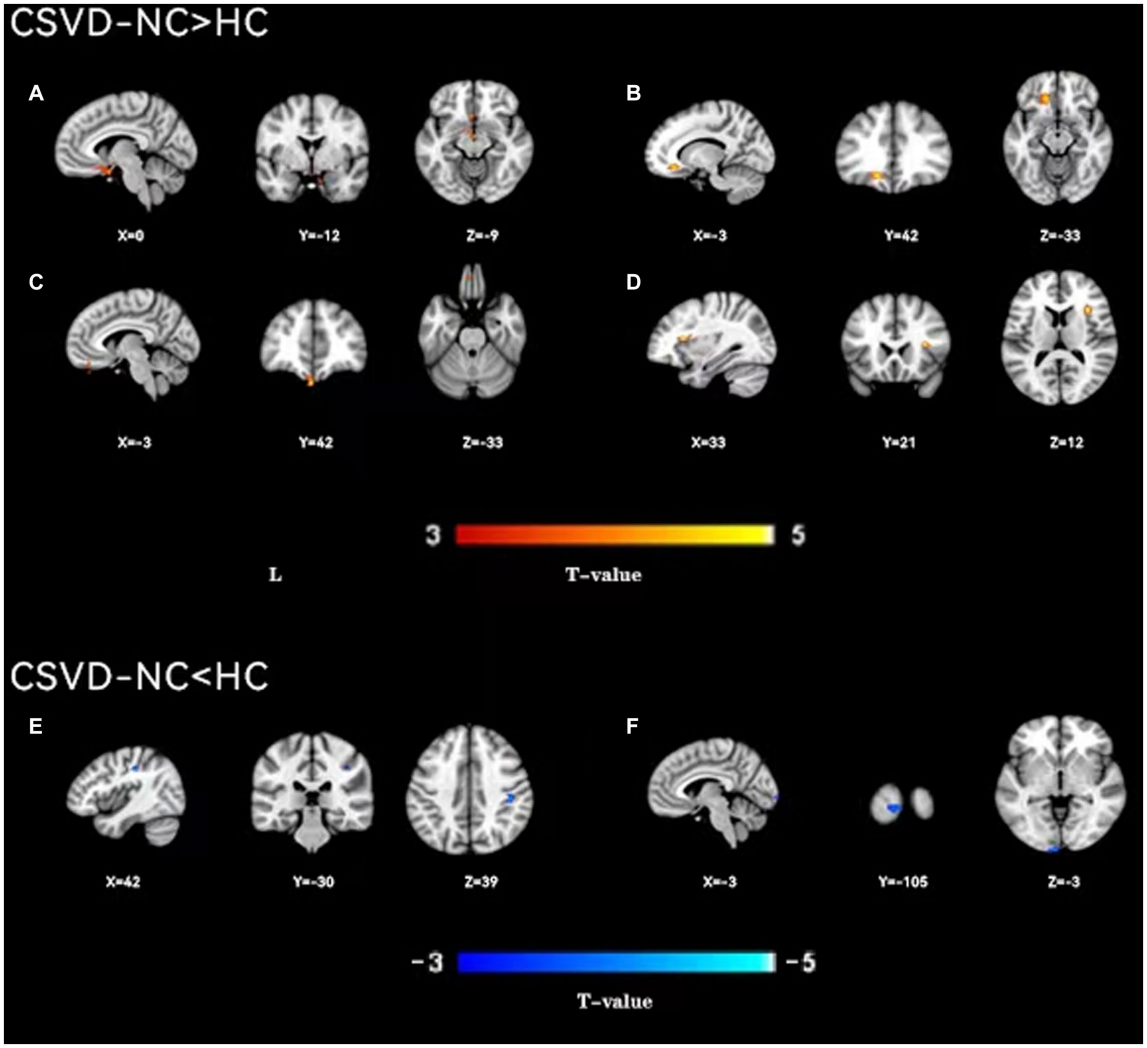
A two-sample *t*-test was used to compare the dynamic ALFF maps between CSVD-NC patients and healthy controls. Significantly increased dynamic ALFF in Left parahippocampal gyrus **(A)**, left orbital middle frontal gyrus **(B)**, left straight gyrus **(C)**, right insular **(D)**; significantly decreased dynamic ALFF in Right postcentral gyrus **(E)** and Left talocortex **(F)**.

As shown in Figure 1, the spatial difference of d-ALFF among the three groups was similar, where the brain regions with low variability were located in the bilateral parietal lobe, frontal lobe, temporal lobe, and occipital lobe, and high variability brain regions were located in the bilateral hippocampus, bilateral cingulate gyrus, and cuneate anterior lobe. Compared with the CSVD group, the d-ALFF values in the frontal lobe, cuneate lobe, precuneate lobe, cingulate gyrus, hippocampus, and parahippocampal gyrus in the HC group were significantly higher, and the overall d-ALFF variability of HCs was higher than the CSVD group. The ANOVA of d-ALFF among the three groups showed that there were significant differences in d-ALFF in the bilateral parahippocampal gyrus, bilateral hippocampus, right precuneus, bilateral insular, right postcentral gyrus, left orbital inferior frontal gyrus, and left straight gyrus among the three groups (Table 3, Figure 2). Further intergroup analysis showed that in the CSVD-CI group, d-ALFF increased in the bilateral parahippocampal gyrus, bilateral hippocampus, left superior orbital frontal gyrus, left middle orbital middle frontal gyrus, left dorsolateral superior frontal gyrus, and right middle orbital frontal gyrus, while d-ALFF decreased in the right postcentral gyrus and left postcentral gyrus (Table 3, Figure 3). Compared with the HC group, the d-ALFF values of the left straight gyrus, left superior orbital frontal gyrus, left orbital middle frontal gyrus, left parahippocampal gyrus, and right insular increased in the CSVD-NC group, while d-ALFF decreased in the right postcentral gyrus (Table 3, Figure 4). CSVD-NC and CSVD-CI did not pass the FWE correction of cluster level between voxels, so we isolated the d-ALFF values of different brain regions among the three groups according to the ANOVA results, and then performed a double sample *t-*test of CSVD-CI and CSVD-NC. It was found that there were significant differences in the bilateral parahippocampal gyrus, bilateral hippocampus, right middle orbital frontal gyrus, and left insular lobe in patients with CSVD (*p* < 0.05 Bonferroni correction, double-tailed test) (Table 4).

**Table 3:** Brain regions with significant differences in d-ALFF among the three groups.

	Brain regions	Size	MNI coordinate	Peak *F*-value
Group effect	ParaHippocampal_L	61	33-39 0	42.22
	ParaHippocampal_R	30	33-39 0	42.22
	Hippocampus_L	52	33-39 0	42.22
	Hippocampus_R	46	33-39 0	42.22
	Frontal_Mid_Orb_R	17	39 48-12	28.66
	Precuneus_R	11	18 0 39	29.31
	Postcentral_R	59	51-9 30	21.45
	Insula_L	13	-36 -3 21	28.03
	Insula_R	15	33 21 12	32.70
	Frontal_Inf_Orb_L	18	-21 27-9	20.87
	Rectus_L	21	0 15-24	30.93
	Calcarine_L	16	-3 -105 -3	20.41

	Brain regions	Size	MNI coordinate	Peak *T* value
CSVD-CI > HC	ParaHippocampal_L	75	24-21 24	6.33
	ParaHippocampal_R	61	24-21 24	6.33
	Hippocampus_L	68	24-21 24	6.33
	Hippocampus_R	57	24-21 24	6.33
	Frontal_Inf_Orb_L	19	-45 42-12	4.52
	Frontal_Mid_Orb_L	11	-18 27 09	4.87
	Frontal_Sup_L	12	-15 72 0	4.23
	Frontal_Mid_Orb_R	21	39 48-12	5.53
CSVD-CI < HC	Postcentral_R	72	51-9 30	5.40
	Postcentral_L	27	-57 -9 39	4.57
CSVD-NC > HC	ParaHippocampal_L	21	0-12 -9	5.74
	Rectus_L	23	-3 42-33	5.35
	Frontal_Sup_Orb_L	11	-3 42-33	5.35
	Frontal_Mid_Orb_L	10	-3 42-33	5.35
	Insula_R	15	33 21 12	5.29
CSVD-NC < HC	Postcentral_R	23	42-30 39	4.19
	Calcarine_L	18	-3 -105 -3	3.94

HC, healthy control; CSVD-NC, cerebral small-vessel Disease without cognitive impairment; MNI, Montreal Neurological Institute; CSVD-CI, cerebral small-vessel Disease with cognitive impairment, R, right. L, left.

**Table 4:** Brain regions with significant differences in d-ALFF between CSVD-CI and CSVD-NC.

Brain regions	Size	MNI coordinate	*t*-value	*p*-value	Adjust *p*-value
ParaHippocampal_L	61	33-39 0	3.738	0.002	0.001
ParaHippocampal_R	30	33-39 0	3.738	0.002	0.001
Hippocampus_L	52	33-39 0	3.738	0.002	0.001
Hippocampus_R	46	33-39 0	3.738	0.002	0.001
Insula_L	13	-36 -3 21	2.992	0.004	0.001

R, Right; L, left; MNI, Montreal Neurological Institute.

### 3.3. Differences in static ALFF

The results of the static ALFF ANOVA showed that there were significant differences in FWE values of the bilateral parahippocampal gyrus, bilateral hippocampus, bilateral cingulate gyrus, bilateral postcentral gyrus, left straight gyrus, left orbital middle frontal gyrus, left triangular inferior frontal gyrus, left dorsolateral superior frontal gyrus, right middle frontal gyrus, right middle temporal gyrus, right middle frontal gyrus, and right orbital inferior frontal gyrus among the three groups (Supplementary Table S1, Supplementary Figure S1). A *t*-test showed that compared with the HC group, the static ALFF of the bilateral hippocampus, bilateral parahippocampal gyrus, left inferior temporal gyrus, and bilateral middle frontal gyrus in the CSVD-CI group increased, while the static ALFF decreased in the left lingual gyrus, right inferior temporal gyrus, bilateral postcentral gyrus, and bilateral cingulate gyrus (Supplementary Table S1, Supplementary Figure S2). Compared with the HC group, the static ALFF values in the bilateral hippocampus, bilateral parahippocampal gyrus, and left orbital middle frontal gyrus in the CSVD-NC group were higher, while those in the right middle temporal gyrus, left postcentral gyrus, right precentral gyrus, bilateral deltoid inferior frontal gyrus, left dorsolateral superior frontal gyrus, and right middle frontal gyrus were lower (Supplementary Table S1, Supplementary Figure S3). CSVD-NC and CSVD-CI did not pass the FWE correction of cluster level at the voxel level, so we extracted the static ALFF values of different brain regions according to the ANOVA and then conducted a double sample *t*-test between the two groups. We found that there was statistical significance in the bilateral hippocampal, bilateral parahippocampal gyrus, right postcentral gyrus, and left dorsolateral superior frontal gyrus in the CSVD-NC and CSVD-CI groups (*p* < 0.05) (Supplementary Table S2).

### 3.4. Correlation analysis

**Figure 5:**
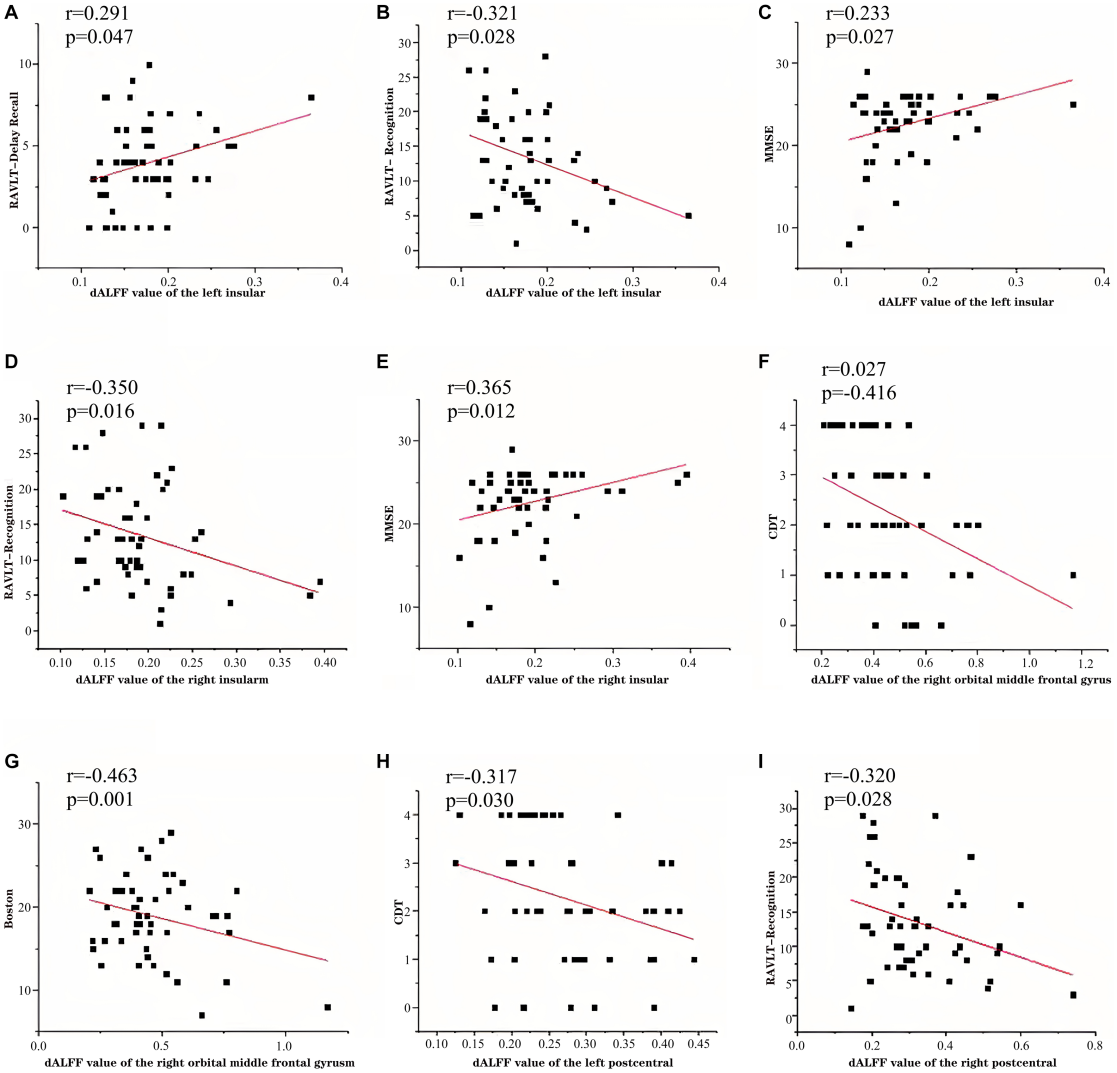
In patients with CSVD-CI, left insular d-ALFF was negatively correlated with RAVLT delayed recall **(A)**, positively correlated with RAVLT recognition **(B)**, positively correlated with MMSE **(C)**, right insular d-ALFF was negatively correlated with RAVLT recognition **(D)**, positively correlated with MMSE **(E)**, right orbital middle frontal gyrus d-ALFF was negatively correlated with CDT **(F)**, negatively correlated with BNT **(G)**, left postcentral gyrus d-ALFF was negatively correlated with CDT **(H)**, and right postcentral gyrus d-ALFF was negatively correlated with RAVLT recognition **(I)**.

The patients with CSVD showed the following clinical correlation. The d-ALFF value of the left insular was positively correlated with the RAVLT delayed recall (*r* = 0.291, *p* = 0.047) (Table 5, Figure 5A), negatively correlated with the RAVLT recognition (*r* = −0.321, *p* = 0.028) (Table 5, Figure 5B), and positively correlated with the MMSE score (*r* = 0.323, *p* = 0.027) (Table 5, Figure 5C). The d-ALFF value of the right insular was negatively correlated with the RAVLT recognition (*r* = −0.350, *p* = 0.016) (Table 5, Figure 5D) and positively correlated with the MMSE score (*r* = 0.365, *p* = 0.012) (Table 5, Figure 5E). The d-ALFF value of the right orbital middle frontal gyrus was negatively correlated with the CDT score (*r* = −0.416, *p* = 0.004) (Table 5, Figure 5F) and negatively correlated with the BNT score (*r* = −0.463, *p* = 0.001) (Table 5, Figure 5G). The d-ALFF value of the left postcentral gyrus was negatively correlated with the CDT score (*r* = −0.317, *p* = 0.030) (Table 5, Figure 5H), and the d-ALFF value of the right postcentral gyrus was negatively correlated with the RAVLT recognition (*r* = −0.320, *p* = 0.028) (Table 5, Figure 5I).

**Table 5:** Difference of d-ALFF between patients with CSVD and HC correlation between brain region and neuropsychological scale.

	Insula_L				Insula_R				Frontal_Mid_Orb_R			
	*r*	*p*	95% CI		*r*	*p*	95% CI		*r*	*p*	95% CI	
			UL	LL			UL	LL			UL	LL
RAVLT												
Delay recall-5 min	0.291	0.047*	0.524	0.022	0.172	0.243	0,468	−0.081	0.131	0.374	0.348	−0.218
Word recognition	−0.321	0.028*	−0.071	−0.529	−0.350	0.016*	−0.079	−0.548	0.047	0.750	0.279	−0.223
CDT	−0.042	0.777	0.210	−0.256	0.106	0.473	0.432	−0.116	−0.416	0.004*	−0.215	−0.604
MMSE	0.323	0.027*	0.555	0.046	0.365	0.012*	0.573	0.028	−0.084	0.571	0.279	−0.366
BNT	−0.009	0.953	0.275	−0.290	0.862	0.026*	0.193	−0.325	−0.463	0.001*	0.051	−0.667

	Postcentral_L				Postcentral_R			
	*r*	*p*	95% CI		*r*	*p*	95% CI	
			UL	LL			UL	LL
RAVLT								
Delay recall-5 min	−0.153	0.299	0.146	−0.442	0.092	0.535	0.340	−0.168
Word recognition	−0.092	0.535	0.162	−0.346	−0.320	0.028*	−0.010	−0.630
CDT	−0.317	0.030*	−0.073	−0.534	−0.160	0.278	0.096	−0.431
MMSE	−0.044	0.766	0.232	−0,278	0.265	0.162	0.454	−0.042
BNT	−0.015	0.918	0.276	−0.281	−0.190	0.196	0.192	−0.501

RAVLT, Rey auditory verbal learning test; CDT, Clock Drawing Test, MMSE, mini mental state examination; BNT, Boston naming test; L, Left, R, Right; 95%CI, 95% confidence interval; UL, Up limit; LL, Low limit.

## 4. Discussion

The present research studied the dynamic brain activity in CSVD using the d-ALFF for the first time. We found that there are dynamic abnormalities of spontaneous brain activity in patients with CSVD, mainly in the bilateral parahippocampal gyrus, bilateral hippocampus, bilateral insular, and frontal lobe. We also showed that functional changes of the bilateral parahippocampal gyrus and bilateral hippocampus were found in the static ALFF and d-ALFF analysis, suggesting that the bilateral parahippocampal gyrus and bilateral hippocampus may be key brain regions in the pathogenesis of CSVD. In addition, the dynamic characteristics of spontaneous activity in multiple brain regions of patients with CSVD were correlated with the neuropsychological scale, suggesting that the dynamic variability of these brain regions may be an index related to CSVD symptoms, which has a certain significance for the evaluation of the disease.

### 4.1. Demographic and clinical characteristics

The prevalence of hypertension (systolic blood pressure) and diabetes in CSVD group was higher than that in HC group, and the prevalence rate of diabetes in CSVD-CI group was higher than that in CSVD-NC group. Previous studies have shown that hypertension and diabetes are important acquired factors of cerebrovascular disease, among which hypertension is the most common, most important and controllable risk factor for stroke (Lin et al., 2015). Studies have shown that controlling blood pressure (mainly reducing systolic blood pressure) can effectively reduce the incidence of stroke. Previous studies have found that there is a difference in the concentration of plasma glycosylated hemoglobin between patients with mild cognitive impairment and normal elderly people, and glycosylated hemoglobin can reflect the blood glucose level of the human body at 2–3 weeks (Cheng et al., 2017). It is worth noting that the score of HAM-A and HAM-D in CSVD-CI is significantly higher than that in the other two groups. Some studies have shown that CSVD can affect the mood of patients and cause apathy, depression, anxiety, etc., and as the disease progresses, the symptoms of anxiety and depression would be aggravated (Feng et al., 2021).

CSVD is an important risk factor to promote the decline of cognitive ability and the early manifestation of dementia. The main reason may be that CSVD causes insufficient cerebral blood supply, leads to local tissue ischemia and hypoxia, and finally leads to changes in brain function, which is characterized by cognitive decline (Kang et al., 2019; Yue et al., 2020). The MMSE score of CSVD patients was significantly lower than that of normal controls. MMSE is a reliable evaluation tool for the diagnosis of cognitive impairment after stroke. Shen and other studies have shown that MMSE in the middle and acute stage can independently predict the functional outcome in the early stage of stroke, which shows that the lower the MMSE score in the early stage of stroke, the worse the prognosis (Shen et al., 2016). The scores of RAVLT immediate recall, delayed recall and recognition in the CSVD group were lower than those in the HC group, indicating that the visual and auditory functions of the patients with cerebral microvascular disease were impaired, and the short-term and long-term memory functions were decreased compared with the normal. The cognitive impairment in the CSVD-CI group was more severe than that in the CSVD-NC group (Gainotti et al., 2008; Kim et al., 2014). It is proved that with the progress of the disease, the visual and auditory impairment worsened gradually, and the decrease of short-term delayed memory score of auditory learning speech test was also observed in AD patients (Kim et al., 2014). BNT test shows that patients with CSVD have different degrees of language impairment. The scores of CDT and TMT test reflect the impairment of execution and computing ability in patients with CSVD, which represents the general decrease of cognitive function (Tuladhar et al., 2016).

### 4.2. Alterations in d-ALFF

We found that the spatial differences of d-ALFF among the three groups were similar. The brain regions with low variability were located in the bilateral parietal lobe, frontal lobe, temporal lobe, and occipital lobe, while the brain regions with high variability were located in the bilateral hippocampus, bilateral cingulate gyrus, and anterior cuneate lobe. The d-ALFF variability in the frontal lobe, cuneate lobe, anterior cuneate lobe, cingulate gyrus, hippocampus, and parahippocampal gyrus in the HC group was significantly higher than in the CSVD group, and the overall d-ALFF variability of HCs was higher than the CSVD group.

The regional brain activity of patients with MCI varies widely, mainly affecting the frontoparietal and temporal lobes. Patients with MCI show extensive and diverse low-frequency amplitude decreases in the frontal, parietal, and temporal lobes, which is confirmed to be related to their cognitive impairment. The bilateral occipital lobe is considered to be the key area of visual image representation, which may explain the impairment of auditory and visual function in patients with CSVD (Tao et al., 2019). The d-ALFF variability in the CSVD group was significantly lower than in the HC group, especially in the frontal lobe, cuneate lobe, anterior cuneate lobe, cingulate gyrus, hippocampus, and parahippocampal gyrus, which are closely related to cognitive function. The frontal lobe, cuneate lobe, anterior cuneate lobe, cingulate gyrus, hippocampus, and parahippocampal gyrus belong to the default mode network (DMN). Extensive studies have shown that the DMN is sensitive to nervous system diseases and can be destroyed in the early stage, which may also be one of the reasons for the decrease in cognitive function in patients with CSVD (Meusel et al., 2019).

There were obvious abnormalities of the d-ALFF in the bilateral hippocampus and parahippocampal gyrus between the three groups. The hippocampus is the processing and integration center of episodic memory in the brain, and the parahippocampal gyrus is the gray layer around the hippocampus, which plays an important role in the coding and extraction of memory (Cheng et al., 2017). The hippocampus and the parahippocampal gyrus are adjacent in anatomical structure, and the ALFF of higher frequencies can be used to clearly represent the synchronization and integration of information in this local structure (Xiao et al., 2018). In patients with MCI, it was confirmed that the increase of the ALFF in the bilateral hippocampus and parahippocampal gyrus represented abnormal hyperactivity of the hippocampus and its adjacent structures, indicating a significant increase in neural activity. Studies have shown that the increased activity of hippocampal neurons is related to the compensatory mechanism of cognitive impairment in patients with hippocampal atrophy (Pan et al., 2017). Neural activities related to the hippocampus and its adjacent structures are significantly enhanced and are used to compensate for the decline in cognitive function associated with long-term memory. Some studies have shown that physical and mental exercise can reduce the abnormal hyperactivity of the hippocampus in patients with MCI, thus improving the cognitive state of patients (Tao et al., 2019). The hippocampus and parahippocampal gyrus belong to the limbic system. Increased d-ALFF variability in the hippocampus and parahippocampal gyrus represents excessive activation of the limbic system, which may emphasize the oversensitivity of CSVD patients to emotional stimuli, especially negative stimulation, which is consistent with a previous study that found that CSVD patients, especially the CSVD-CI group, were significantly more anxious than the other two groups (Cui et al., 2020).

This study showed that there were significant differences in the bilateral insular d-ALFF. The insular plays an important role in social, emotional, autonomy, and motor control, risk prediction, and decision-making (Menon and Uddin, 2010). The human insular can be divided into three regions, the dorsal anterior insula cortex (dAIC), ventral anterior insula cortex (vAIC), and posterior insula cortex (PIC). The dAIC region connects to the frontal, anterior cingulate, and parietal areas, and is related to cognitive control processes. The PIC, which is connected with brain regions, participates in sensorimotor processing, and the vAIC connects with marginal regions for emotional processing (Chang et al., 2013). Insular volume atrophy has been observed in patients with previous MCI. It is known that the cognitive impairment of subcortical vascular dementia may be related to the interruption of the cholinergic pathway through subcortical white matter (Ni et al., 2016; Cheng et al., 2017). It is well known that the dysfunction of the cholinergic pathway can adversely affect short-term episodic memory, attention, and executive function. In addition, the peripancreatic branches of the lateral cholinergic pathway originate from the basal forebrain and project to the tegmental region, including the superior temporal gyrus and insular cortex (Selden et al., 1998; Bocti et al., 2005). Our clinical correlation study showed that in patients with CSVD, the d-ALFF value of the left insular was positively correlated with the RAVLT delayed memory score and MMSE score and negatively correlated with the RAVLT recognition score. The activation degree of the bilateral insular is related to the cognitive function of patients, and may be related to the destruction of this lateral cholinergic pathway. At the same time, the left insular is significantly related to short-term episodic memory, which can also explain the over-activation of insular function. Short-term episodic memory was decreased in patients. Similar to this study, Xiong et al. found that the local consistency of the left insular was increased in patients with type 2 diabetes (Xiong et al., 2020). Therefore, we conclude that the increase of insular d-ALFF may be one of the early compensatory mechanisms in patients with CSVD cognitive impairment.

Among the three groups, the d-ALFF of the right precuneus was increased, and the precuneus was located on the medial side of the parietal lobe, which was related to a higher level of cognitive function, such as episodic memory, self-related information processing, and all aspects of consciousness. The precuneus is considered to be the center of the central executive network of the frontal parietal lobe. The prefrontal lobe is usually activated during the recovery of episodic memory, and this activation is related to visual images in the process of memory (Ni et al., 2016; Su et al., 2019). In addition, some studies have shown that the volume of the anterior wedge lobe is related to anterograde memory impairment caused by posterior cortical atrophy (Li et al., 2017; Yu et al., 2019). In previous frontal cortex studies, the regional coherence/synchronization of cuneiform leaves decreased. The increased spontaneous activity of the anterior cuneus may be due to the increased local abnormal activity in patients with early cognitive impairment, which belongs to the compensatory mechanism of cognitive impairment. Our experiments have shown that patients with CSVD have changes in executive function and attention.

An extensive increase of the d-ALFF in the frontal lobe was found between the three groups. The DMN includes the medial prefrontal cortex, anterior cuneate lobe, cuneate lobe, posterior cingulate cortex, and parahippocampal gyrus, which play an important role in internal process monitoring and memory extraction (Tao et al., 2019). Cognitive function, including executive function and processing speed, depends on the integrity of the frontal-subcortical and frontoparietal networks. We found that most of the brain regions with high variability of d-ALFF were located in the DMN, such as the left superior orbital frontal gyrus, left orbital middle frontal gyrus, left inferior orbital frontal gyrus, left dorsolateral superior frontal gyrus, left straight gyrus, right orbital middle frontal gyrus, bilateral hippocampus, bilateral parahippocampal gyrus, and precuneate lobe. A number of studies have shown that frontal lobe dysfunction is the most typical model of cerebral microvascular disease, and the frontal lobe is the most severely damaged area in patients with cognitive impairment (Zhang et al., 2011; Niendam et al., 2012; Tuladhar et al., 2015; Dickie et al., 2016). Studies have shown that the frontal lobe of patients with cognitive impairment shows significant atrophy of the bilateral superior orbital gyrus gray matter volume. The orbitofrontal gyrus is the key node of the reward system, and there is significant damage to the reward system of CSVD patients. Cognitive impairment in patients with CSVD is associated with decreased frontal cortex connectivity in the bilateral frontal lobes, and decreased frontal cortex connectivity mediates frontal lobe volume atrophy and frontal lobe-related executive dysfunction caused by cerebral microvascular disease (Lawrence et al., 2014; Lei et al., 2016). Studies in patients with Alzheimer’s and MCI have shown that increased prefrontal lobe activation during specific cognitive tasks or increased frontal lobe activity at rest is considered to be a compensatory reallocation of cognitive resources (Li et al., 2019). The increase in d-ALFF variability indicates abnormal temporal fluctuations in local brain activity in the frontal lobe. This abnormality may impair the ability of the frontal lobe network to regulate cognitive function in patients with CSVD, resulting in cognitive decline. At the same time, we found that the d-ALFF variability of the right orbital middle frontal gyrus was negatively correlated with the CDT score and BNT score, which also confirmed that the overactivation of the right orbital middle frontal gyrus might be the compensatory mechanism of executive and cognitive function.

The value of the d-ALFF in the right postcentral gyrus in the CSVD group was lower than in the HC group. Studies have shown that the postcentral gyrus is a key area in the somatosensory network and participates in daily activities (Fu et al., 2019). The somatosensory network plays an important role in execution and action recognition, is regarded as one of the key brain regions in motion vision processing, and controls the learning of early motor skills acquisition (Bernardi et al., 2015). The decrease of the d-ALFF in the postcentral gyrus indicates that the integrity of the somatosensory network is impaired, which is consistent with previous studies that found that the postcentral gyrus in MCI and Alzheimer’s patients have motor sensory disorders (Kavcic et al., 2011).

### 4.3. Alterations in static ALFF

Similar to the d-ALFF, there were significant changes in the bilateral hippocampal and hippocampal static ALFF among the three groups, which further confirmed that there was a significant correlation between the bilateral hippocampal and parahippocampal gyrus and cognitive decline in patients with CSVD. Compared with the d-ALFF analysis, CSVD patients showed more extensive static ALFF changes in the frontal lobe and decreased static ALFF values in several frontal lobe regions, such as the bilateral inferior triangular frontal gyrus, left dorsolateral superior frontal gyrus, right middle frontal gyrus, and left lingual gyrus, indicating that the frontal lobe was widely activated in CSVD patients and played an important role in cognitive impairment. However, the changes of static ALFF in the frontal lobe were more diverse than those in the d-ALFF. Perhaps because the d-ALFF takes into account the influence of spontaneous brain activity, static ALFF and d-ALFF complement each other, which can help better understand the neurophysiological mechanism of CSVD. Similar to the d-ALFF, CSVD patients showed a decreased static ALFF in the postcentral gyrus, indicating that the postcentral gyrus is an important brain area in the pathogenesis of CSVD.

Different from the d-ALFF, static ALFF in the right middle temporal gyrus in the CSVD-NC group was lower than the HC group, and the static ALFF in the left middle temporal gyrus in the CSVD-CI group was lower than the HC group. Wang and other studies have shown that the infratemporal gyrus is considered to be the central part of the language-forming area, known as the tertiary visual associative cortex, and is also involved in visual perception, language, and memory (Wang J. et al., 2019; Wang P. et al., 2019). According to previous reports, both Alzheimer’s and MCI patients showed a decrease in the thickness of the temporal lobe cortex, which significantly affected the memory function of the patients (Ni et al., 2016). The static ALFF value of the bilateral cingulate gyrus in the CSVD-CI group was significantly lower than in the HC group. Previous studies have found atrophy of the cingulate cortex in patients with Alzheimer’s. The cingulate gyrus has been shown to be related to emotion and cognition. As an important part of the DMN, the posterior cingulate gyrus completes the process of learning and memory through the detection and motivation of behavior (Heilbronner and Platt, 2013). A posterior cingulate gyrus injury has been shown to cause learning and memory impairment. The decreased activation of the bilateral cingulate gyrus in the CSVD-CI group may also be one of the causes of cognitive impairment in CSVD patients. It is worth mentioning that compared with HCs, the CSVD-NC group showed a decrease in the value of static ALFF in the bilateral hippocampus and parahippocampal gyrus, which may be due to the early course of CSVD. The patients did not show functional compensation of the hippocampus and parahippocampal gyrus, which suggests that the spontaneous activity of the brain was related to the course of the disease.

### 4.4. Limitations

The present study has some limitations. First, the present study investigated CSVD patients in a wide frequency range (0.01–0.08 Hz) of ALFF and d-ALFF changes, but this low-frequency range can be further divided into several different frequency bands, such as Slow-6 (0–0.01 Hz), Slow-5 (0.01–0.027 Hz), Slow-4 (0.027–0.073 Hz), Slow-3 (0.073–0.198 Hz), and Slow-2 (0.198–0.25 Hz). The present study did not focus on the d-ALFF values in different frequency bands. Second, the present study only focused on the local dynamic brain function abnormalities in patients with CSVD and did not focus on the dynamic whole-brain functional connectivity abnormalities due to further study at a later stage. Finally, among the local brain function indicators, the present paper only focused on the d-ALFF and did not focus on the changes in dynamic local coherence (regional homogeneity); combining the two may be able to better understand the changes in local dynamic brain activity in patients with CSVD. In conducting the correlation study, partial correlation analysis was used in order to exclude the effects of gender, age, and years of education, but some of the variables may be non-parametric and the results may still have small biases despite multiple tests. This study is a cross-sectional small sample study, and it is necessary to increase the sample size and longitudinally observe the disease progress of the patients.

## 5. Conclusion

Studies have shown that there are dynamic abnormalities of brain spontaneous activity in patients with CSVD, mainly in bilateral parahippocampal gyrus, bilateral hippocampus, bilateral insular and frontal lobe. The functional changes of bilateral parahippocampal gyrus and bilateral hippocampus were found in sALFF and d-ALFF analysis, suggesting that bilateral parahippocampal gyrus and bilateral hippocampus may be the key brain regions in the pathogenesis of CSVD. The dynamic characteristics of spontaneous activity in multiple brain regions of patients with CSVD are correlated with the neuropsychological scale, suggesting that the dynamic variability of these brain regions may be an index related to CSVD symptoms, which has a certain significance for the evaluation of the disease.

## Data availability statement

The original contributions presented in the study are included in the article/Supplementary material, further inquiries can be directed to the corresponding author.

## Ethics statement

The studies involving human participants were reviewed and approved by the Research Ethics Committees of the Affiliated Hospital of North Sichuan Medical College. The patients/participants provided their written informed consent to participate in this study.

## Author contributions

JRS, TL, and LC contributed equally to the experiments, data analysis, writing and revising the manuscript. LJZ, YjL, and WY contributed to performing the experiments and data analysis. HQL contributed to the data collection. All authors contributed to the article and approved the submitted version.

## Funding

This study was supported by The National Clinical Key Specialty Construction Manuscript (Sichuan Provincial Health Commission Medical Policy Letter [2023] No. 87),andthe Sichuan Science and Technology Program (2019YJ0380) and Nanchong Science and Technology Program (20SXZRKX0011).

## Conflict of interest

The authors declare that the research was conducted in the absence of any commercial or financial relationships that could be construed as a potential conflict of interest.

## Publisher’s note

All claims expressed in this article are solely those of the authors and do not necessarily represent those of their affiliated organizations, or those of the publisher, the editors and the reviewers. Any product that may be evaluated in this article, or claim that may be made by its manufacturer, is not guaranteed or endorsed by the publisher.

## Footnotes

^1^ http://rfmri.org/dpabi
