# Supplementary material for: Dynamic alterations in the amplitude of low-frequency fluctuation in patients with cerebral small vessel disease
Source: Front Mol Neurosci. 2023 Sep 22;16:1200756. doi: 10.3389/fnmol.2023.1200756 (PMC10556663; doi:10.3389/fnmol.2023.1200756)
Supplement: Supplementary file 1 [file Data_Sheet_1.docx]

TableS1 :Brain regions with significant differences in sALFF among the three groups

|  | Brain regions | | Size | MNI coordinate | Peak F value |
| --- | --- | --- | --- | --- | --- |
| Group effect | ParaHippocampal_L | | 79 | -33 -35 6 | 49.22 |
|  | ParaHippocampal_R | | 66 | -33 -35 6 | 49.22 |
|  | Hippocampus_L | | 92 | -33 -35 6 | 49.22 |
|  | Hippocampus_R | | 85 | -33 -35 6 | 49.22 |
|  | Rectus_L | | 10 | 0 63 -15 | 24.75 |
|  | Frontal_Mid_Orb_L | | 18 | 0 63 -15 | 24.75 |
|  | Lingual_L | | 76 | -12 -54 -9 | 21.25 |
|  | Temporal_Inf_R | | 12 | 45 -63 -3 | 19.11 |
|  | Temporal_Mid_R | | 23 | 45 -63 -3 | 19.11 |
|  | Cingulum_Post_R | | 72 | 6 -24 27 | 32.41 |
|  | Cingulum_Post_L | | 85 | 6 -24 27 | 32.41 |
|  | Postcentral_L | | 137 | -54 -6 42 | 36.99 |
|  | Postcentral_R | | 253 | 54 -9 30 | 39.29 |
|  | Parietal_Inf_R | | 68 | 51 -33 51 | 22.23 |
|  | Frontal_Mid_R | | 46 | 30 -3 54 | 26.92 |
|  | Paracentral_Lobule_L | | 59 | -12 -24 69 | 28.51 |
|  | Paracentral_Lobule_R | | 65 | 51 -26 51 | 42.66 |
|  | Temporal_Mid_R | | 43 | 51 -30 -9 | 21.25 |
|  | Frontal_Inf_Orb_R | | 65 | 54 21 27 | 32.40 |
|  | Frontal_Inf_Tri_L | | 36 | -33 24 24 | 31.56 |
|  | Frontal_Inf_Tri_L | | 35 | -24 36 45 | 25.04 |
|  | Brain regions | Size | | MNI coordinate | t value |
| CSVD-CI>HC | Temporal_Inf_L | 50 | | -36 -45 0 | 7.46 |
|  | Hippocampus_L | 125 | | -36 -45 0 | 7.46 |
|  | Hippocampus_R | 104 | | -36 -45 0 | 7.46 |
|  | ParaHippocampal_L | 127 | | -36 -45 0 | 7.46 |
|  | ParaHippocampal_R | 95 | | -36 -45 0 | 7.46 |
|  | Frontal_Mid_Orb_L | 18 | | 3 66 -12 | 4,71 |
|  | Frontal_Mid_Orb_R | 10 | | 3 66 -12 | 4,71 |
| CSVD-CI<HC | Lingual_L | 79 | | -12 54 -9 | 4.48 |
|  | Temporal_Inf_R | 14 | | 45 -63 -3 | 4.21 |
|  | Postcentral_R | 250 | | 54 -9 30 | 6.15 |
|  | Postcentral_L | 140 | | -54 -6 42 | 6.32 |
|  | Cingulum_R | 77 | | 9 -15 42 | 5.33 |
|  | Cingulum_L | 103 | | 9 -15 42 | 5.33 |
|  | Paracentral_Lobule_R | 81 | | 51 -42 51 | 4.66 |
| CSVD-NC>HC | ParaHippocampal_L | 74 | | 15 3 30 | 6.30 |
|  | ParaHippocampal_R | 41 | | 15 3 30 | 6.30 |
|  | Hippocampus_L | 64 | | 15 3 30 | 6.30 |
|  | Hippocampus_R | 43 | | 15 3 30 | 6.30 |
|  | Frontal_Mid_Orb_L | 38 | | -3 66 -6 | 5.66 |
| CSVD-NC<HC | Lingual_L | 122 | | -21 -69 -9 | 4.61 |
|  | Temporal_Mid_R | 49 | | 54 -30 -12 | 4.55 |
|  | Temporal_Inf_R | 45 | | 45 -66 -3 | 4.01 |
|  | Postcentral_L | 55 | | 54 -3 42 | 5.07 |
|  | Precentral_R | 80 | | 42 6 30 | 4.84 |
|  | Frontal_Inf_Tri_R | 64 | | 54 21 27 | 5.17 |
|  | Frontal_Inf_Tri_L | 37 | | -33 24 24 | 5.70 |
|  | Frontal_Sup_Medial_R | 13 | | 3 21 42 | 4.45 |
|  | Frontal_Sup_Medial_R | 41 | | -24 39 42 | 4.98 |
|  | Frontal_Mid_R | 84 | | 30 9 51 | 5.51 |

HC, healthy control;CSVD-NC, cerebral small-vessel Disease without cognitive impairment; MNI, Montreal Neurological Institute;CSVD-CI, cerebral small-vessel Disease with cognitive impairment, R, right. L, left.

TableS2: Brain regions with significant differences in sALFF between CSVD-CI and CSVD-NC

| Brain regions | Size | MNI coordinate | t value | P value | Adjust P value |
| --- | --- | --- | --- | --- | --- |
| Hippocampus_L | 92 | -33 -45 6 | 2.836 | 0.007 | 0.010 |
| Hippocampus_R | 85 | -33 -45 6 | 2.836 | 0.007 | 0.010 |
| ParaHippocampal_L | 79 | -33 -45 6 | 2.836 | 0.007 | 0.010 |
| ParaHippocampal_R | 66 | -33 -45 6 | 2.836 | 0.007 | 0.010 |
| Postcentral_R | 253 | 54 -9 30 | -3.738 | 0.001 | 0.041 |
| Frontal_Sup_L | 24 | -24 36 45 | 2.924 | 0.005 | 0.031 |

L, left; R,right; MNI, Montreal Neurological Institute


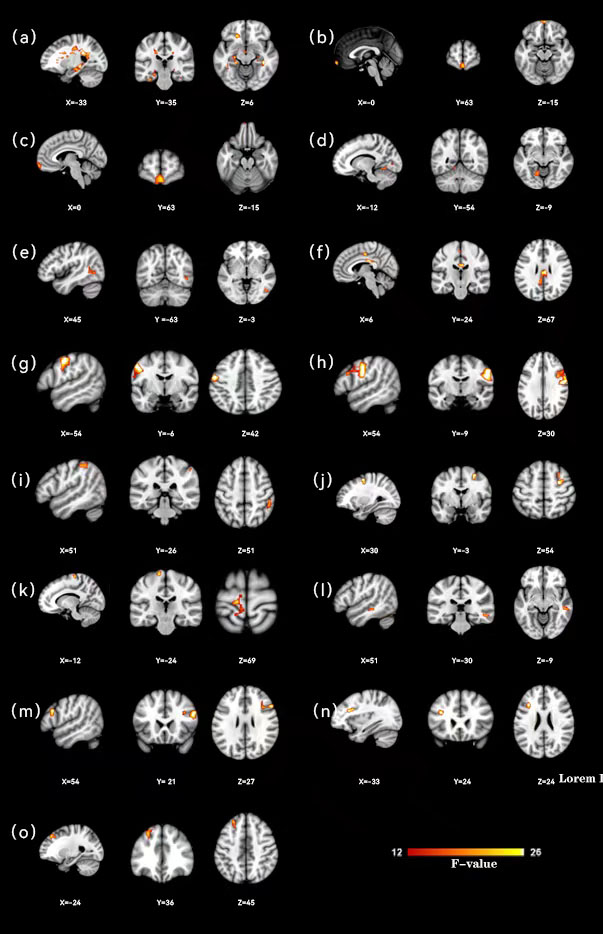


FigureS1:Change static ALFF among three groups. Three groups show static ALFF(dALFF) widespread differences were present predominantly in the Bilateral hippocampus and parahippocampal gyrus(a), Left orbital middle frontal gyrus(b),Left straight gyrus(c), Left lingual gyrus(d), Right middle temporal gyrus(e), Bilateral cingulate gyrus(f), Left postcentral gyrus(g), Right postcentral gyrus(h), Right paracenter lobule(i), Right middle frontal gyrus(j), left paracenter lobule(k), Right middle temporal gyrus(l), Right orbital inferior frontal gyrus (m), Inferior frontal gyrus of left triangle (n), Left dorsolateral superior frontal gyrus (o). Bilateral hippocampus and parahippocampal gyrus


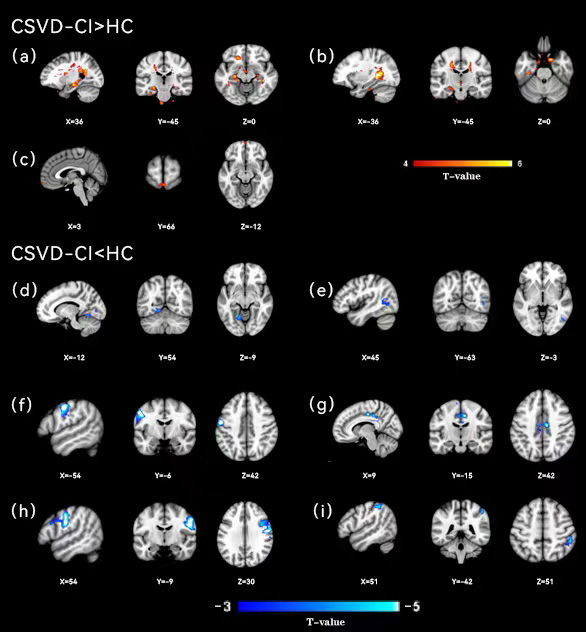


FigureS2:A two-sample t-test was used to compare the static ALFF maps between CSVD-CI patients and healthy controls.Significantly increased static ALFF in Bilateral hippocampus and parahippocampal gyrus(a), Left inferior temporal gyrus(b), Bilateral orbital middle frontal gyrus(c); significantly decreased static ALFF in Left lingual gyrus(d),. Right inferior temporal gyrus(e), Left postcentral gyrus(f),Bilateral cingulate gyrus(g), Right postcentral gyrus(h), Right paracenter lobule(i).


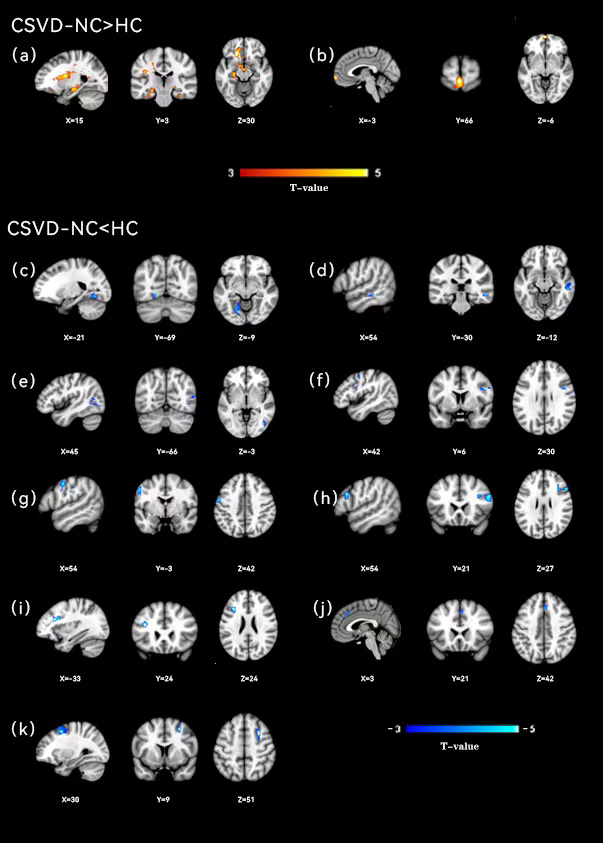


FigureS3:A two-sample t-test was used to compare the static ALFF maps between CSVD-NC patients and healthy controls.Significantly increased static ALFF in Bilateral hippocampus and parahippocampal gyrus(a), Left orbital middle frontal gyrus(b); significantly decreased static ALFF in Left lingual gyrus(c), Right middle temporal gyrus(d),. Right inferior temporal gyrus(e), Right precentral gyrus(f), Left postcentral gyrus(g), Inferior frontal gyrus of right triangle(h), Inferior frontal gyrus of left triangle(i),Right medial superior frontal gyrus(j), Right middle frontal gyrus(k).
